# Molecular Profiling of Endometrial Malignancies

**DOI:** 10.1155/2010/162363

**Published:** 2010-03-28

**Authors:** Norasate Samarnthai, Kevin Hall, I-Tien Yeh

**Affiliations:** ^1^Department of Pathology, University of Texas Health Science Center at San Antonio, San Antonio, TX 78229, USA; ^2^Department of Obstetrics and Gynecology, University of Texas Health Science Center at San Antonio, San Antonio, TX 78229, USA

## Abstract

Molecular profiling of endometrial neoplasms reveals genetic changes in endometrial carcinomas that support the dualistic model, in which type I carcinomas are estrogen-dependent, low grade lesions and type II carcinomas are nonestrogen dependent and high grade. The molecular changes in type I endometrial carcinomas include mutations in *PTEN, PIK3CA, KRAS,* and *β*-catenin, along with microsatellite instability, whereas type II endometrial carcinomas are characterized by genetic alterations in *p53, HER2/neu, p16,* and E-cadherin. For endometrial neoplasms with a malignant mesenchymal component, *C-MYC* mutations and loss of heterozygosity are frequently seen in carcinosarcomas, and a fusion gene, *JAZF1/JJAZ1*, is distinctive for endometrial stromal sarcoma. In addition, *p53* mutations may play an important role in tumorigenesis of undifferentiated endometrial sarcoma. These molecular changes can help in the diagnosis of endometrial neoplasms, as well as form the basis of molecular targeted therapy.

## 1. Introduction

Endometrial malignancies can be categorized into two main groups based on the cell of origin: (i) endometrial carcinoma including carcinosarcoma and (ii) endometrial stromal sarcoma. Endometrial carcinomas show a broad spectrum of phenotypes which show various histologic appearances for example, endometrioid, serous, mucinous, squamous, urothelial, or clear cell, reflecting the differentiation potential of the müllerian epithelium and the difference in the tumorigenetic pathways of each tumor type. Women with an inherited predisposition for endometrial neoplasm have been reported, associated with autosomal dominant disorders such as hereditary nonpolyposis colorectal carcinoma (HNPCC) and Cowden syndrome. Some endometrial carcinomas undergo mesenchymal differentiation and are termed carcinosarcomas (formerly termed malignant mixed müllerian tumors). Pathogenetically and clinically, two distinct forms of endometrial adenocarcinoma, type I and type II, have been described. The molecular alterations driving endometrial carcinogenesis may follow a sequence similar to Vogelstein's model for the progression of colorectal adenoma to carcinoma. This process is accompanied by stepwise genetic changes of oncogenes and tumor suppressor genes. Endometrial stroma may give rise to neoplasms that resemble normal endometrial stromal cells. The spectrum of endometrial stromal tumors ranges from the benign stromal nodule to the malignant endometrial stromal sarcoma. An oncogenic fusion gene, *J* *A* *Z* *F*1/*J* *J* *A* *Z*1 plays a significant role in tumor development of endometrial stromal sarcomas [1].

## 2. Molecular Profiling of Endometrial Carcinoma

### 2.1. Dualistic Model of Endometrial Tumorigenesis

Endometrial carcinoma is the most common malignant neoplasm of female genital tract in developed countries [2] with an estimated 42,160 new cases diagnosed in the United States for 2009 [3]. Approximately 90% of cases of endometrial carcinoma are sporadic, whereas the remaining 10% of cases are hereditary [4]. Clinically, the patients with endometrial carcinomas most often present with abnormal uterine bleeding. In advanced stages, patients may complain of pelvic pain, reflecting spread of the carcinoma. Bokhman [5] first described the pathogenetic classification of 2 different types of endometrial carcinoma, designated as type I and type II carcinomas, according to the determination of biological properties of the tumor, its clinical course, and the prognosis of the disease. 

#### 2.1.1. Type I (Endometrioid Endometrial Carcinoma) [1, 2, 4–13]

**Figure 1:**
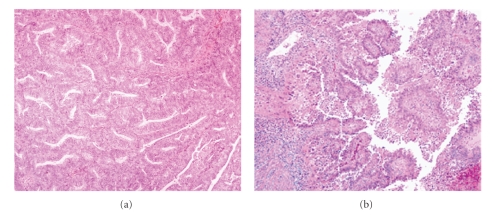
The prototypes for the dualistic model of endometrial carcinoma. Type I endometrioid endometrial carcinoma shows glands lined by stratified neoplastic columnar cells (a), ×100; and type II serous carcinoma showing papillary structures and high nuclear grade (b), ×100.

Type I carcinomas represent the majority of sporadic cases of endometrial carcinoma, accounting for 70–80% of new cases [4, 9–12] which occur predominantly in pre- and perimenopausal women. These cancers are typically of endometrioid type (Figure 1(a)). Risk factors include obesity, hyperlipidemia, and hyperestrogenism for example, anovulation, nulliparity/infertility, late onset of menopause, and endometrial hyperplasia. The tumors in this category are generally low-grade, low-stage, and indolent. They commonly express estrogen and progesterone receptors [2, 4–11]. The rare mucinous carcinomas are also considered type I carcinomas because they usually express estrogen and/or progesterone receptors and are of low histologic grade [10, 11].

#### 2.1.2. Type II (Nonendometrioid Endometrial Carcinoma) [1, 2, 4–13]

Type II carcinomas are less common, accounting for 10–20% of endometrial carcinoma [4, 6]. They are nonendometrioid in differentiation, most frequently papillary serous (Figure 1(b)) and less frequently clear cell, have high-grade histology, typically arise in an atrophic endometrial background, and often have deep myometrial penetration. They usually occur at an older age, approximately 5–10 years later than type I tumors. There is no relationship to estrogen stimulation. Clinically, type II cancers have an aggressive behavior, with a high frequency of distant spread to pelvic lymph nodes. Small cell, undifferentiated and squamous cell carcinomas may also be encountered among type II carcinomas, but little is known about their tumorigenesis [11]. The clinical and pathological features of the two types of endometrial carcinomas are summarized in Table 1. 

### 2.2. Common Molecular Genetic Alterations in Dualistic Model

Evidence for divergent molecular alterations supporting the dualistic model of endometrial tumorigenesis became available approximately 10 years after Bokhman's description of the clinical and pathologic features. The two distinct histological types of carcinomas are associated with genetic alterations of independent sets of genes. These genetic changes may occur singly or in various combinations which differ between individual cases [9]. 

Westin and colleague [14] described that expression of estrogen-induced genes, *RALDH2, EIG121, SFRP1, SFRP4, IGF-1*, and *IGF-IR*, tend to be highest in the well-to-moderately differentiated endometrioid carcinoma. This finding supports the partitioning of endometrial carcinoma into two distinct groups by traditional estrogen-related classification. According to this model, normal endometrial cells would transform into endometrioid endometrial carcinoma through 5 different molecular changes, including, mutations of *PTEN, PIK3CA, KRAS*, and *CTNNB1* (*β*-catenin) genes and microsatellite instability (MSI) while non-endometrioid endometrial carcinoma is frequently related to alterations of *p*53 and chromosomal instability [7, 8, 15, 16]. Non-endometrioid endometrial carcinoma frequently demonstrates high-ordered aneuploidy and has an intact mismatch repair (MMR) mechanism [12]. Furthermore, none of the five main alterations of endometrioid endometrial carcinoma (mutations of *PTEN, PIK3CA, KRAS*, and *CTNNB1* genes and MSI) plays a major role in non-endometrioid endometrial carcinoma. However, in many endometrial carcinomas exhibit overlapping clinical, morphologic, immunohistochemical, and molecular features of the both types of carcinoma for example, a subset of endometrioid endometrial carcinoma is found with a background of atrophic endometrium or papillary serous carcinoma may occasionally develop from a pre-existing endometrioid endometrial carcinoma and may share histological and genetic features [8–10]. Matias-guiu et al. [8] described the development of non-endometrioid endometrial carcinoma through these possible pathways: (i) *de novo*, through *p*53 mutations, loss of heterozygosity (LOH) at several loci, and some other still unknown gene alterations; or (ii) through dedifferentiation of a pre-existing endometrioid carcinoma. These dedifferentiated non-endometrioid endometrial carcinomas exhibit overlapping features with type I endometrioid endometrial carcinoma [8]. 

Comparison of the major genetic alterations between type I and type II endometrial carcinomas is shown in Table 2. 

Molecular genetic alterations have been extensively investigated in endometrioid and papillary serous adenocarcinomas of the endometrium. These two tumor types are characterized by distinctive molecular alterations, and their tumorigenesis follow separate pathways. 

### 2.3. Molecular Pathology of Endometrioid Carcinomas

#### 2.3.1. PTEN

The most frequently altered gene in endometrioid endometrial carcinoma is *PTEN* (phosphatase and tensin homologue deleted from chromosome 10), also called *MMAC1* (mutated in multiple advanced cancers 1).* PTEN* behaves as a tumor suppressor gene, is located on chromosome 10q23.3 and encodes a lipid phosphatase that antagonizes the PI3K/AKT pathway by dephosphorylating PIP3, the product of PI3K. This lipid molecule is an important second messenger that regulates the phosphorylation of a protein termed AKT, also known as protein kinase B. Decreased *PTEN *activity causes increased cell proliferation and survival through modulation of signal transduction pathways. 

*PTEN* may be inactivated by several mechanisms such as mutation, LOH, and promoter hypermethylation. Somatic *PTEN* mutations are common in endometrial carcinoma, and they are almost exclusively restricted to endometrioid endometrial carcinomas, occurring up to 83% of them [1, 4, 11, 12]. Germline mutations of *PTEN* are responsible for Cowden syndrome [9, 12]. *PTEN* may be also inactivated by deletion, as shown by LOH in 40% of endometrial carcinomas [7–9, 17]. Promoter hypermethylation leading to *PTEN* inactivation, is found in about 20% of tumors, most of which are high-stage [10]. 

*PTEN* mutations have been detected in 15–55% of endometrial hyperplasias with and without atypia [9, 13]. Interestingly, concordance between MSI status and *PTEN *mutations has been found; the mutations occur in 60–86% of MSI-positive endometrioid endometrial carcinoma but in only 24–35% of the MSI-negative cases [7–9, 13, 17]. This suggests that *PTEN* could be a target for mutations in the context of DNA repair deficiency [13]. In addition, identical *PTEN* mutations have been also identified in hyperplasias coexisting with MSI-positive endometrioid endometrial carcinoma, which suggests that *PTEN* mutations are early events in their development [8]. On the other hand, identical *PTEN* mutations have been detected in MSI-negative endometrial hyperplasia with coexisting MSI-positive endometrioid endometrial carcinomas. Thus, some *PTEN *mutations may precede MSI, and coexistence of both alterations does not necessarily mean a cause-effect relationship [9]. Evaluation of *PTEN* inactivation in endometrial carcinoma precursor lesions by *PTEN* immunostaining has been proposed. However, commercially available antibodies (e.g., clone 10P03, 28H6, polyclonal, 6H2.1) do not have statistically significant associations with the molecular genetic alterations [7, 9, 19]. Some data suggest that *PTEN* is associated with younger age, low stage, endometrioid histology, low histologic grade, and favorable prognosis (78% 5-year survival for patients without mutations, compared with 95% and 93% for patients with one or more mutations, resp.) [7, 9]. In addition, recent data suggest that only *PTEN* mutations outside exons 5–7 may predict favorable survival, independent of the clinical and pathological features of the tumors [9].

#### 2.3.2. PIK3CA

The *PIK3CA* (p110*α* catalytic subunit of PI3K) gene locates on chromosome 3q26.32. Phosphatidylinositol-3-kinase (PI3K) is heterodimeric lipid kinase consisting of a catalytic subunit (p110) and a regulatory subunit (p85) in PI3K/AKT signaling pathway. This pathway is frequently activated in endometrial carcinoma through various genetic alterations and their combinations. Activation of PI3K produces the second messenger PIP3 which subsequently activates various down-stream pathways such as AKT. This regulation involves suppression of apoptosis and enhancement of cell proliferation [9]. *PIK3CA* activation is reported in 26–36% of endometrial carcinoma and may coexist with *PTEN* (15–27%) [7, 9, 12, 15, 20] and *KRAS* mutations [9, 15, 20] suggesting that the *PIK3CA* mutations cooperate with these alterations in malignant transformation [16]. Mutations in *AKT* family members and their correlation with other gene alterations are found in endometrial carcinoma, including *AKT2* (D399N), *AKT2* (D32H) and *AKT3* (E438D) mutations. Mutations of *AKT3* (E438D) also have amplification of and a mutation in* PIK3CA *[21]. *AKT1 E17K* mutation is not associated with either *PTEN* or *PIK3CA* genomic alteration [21]. In vitro studies showed that activating mutations of *PIK3CA* in combination with *PTEN* mutations led to an additional increase in phosphorylated *AKT* when compared with cells with only inactivated *PTEN* [6]. Some investigators have claimed that *PIK3CA* mutations are mutually exclusive of *PTEN* mutations, suggesting that tumorigenic signaling through this pathway can occur either through activation of *PIK3CA* or inactivation of *PTEN* [9]. Recently, interactions between the *PI3K/AKT* and *p53* signaling pathways have been described in which activation of the *PI3K/AKT* pathway through *PTEN* or *PIK3CA* mutations, together with *p53* inactivation, results in malignant transformation [15]. Moreover, patients with dysregulation of PI3K/AKT signaling pathway and *p53* alterations had shorter survival than patients with only *p53* alterations [15]. Mutations were more common in mixed endometrioid-nonendometrioid adenocarcinomas (44%) than in pure endometrioid adenocarcinomas (28%) or pure nonendometrioid adenocarcinomas (21%) [15]. In fact, *PIK3CA* mutations are usually missense and cluster in exons 9 (helical domain) and 20 (kinase domain). The tumors carrying exon 9 *PIK3CA* mutations are more likely to be low-grade carcinomas; in contrast, carcinomas with exon 20 mutations or *PIK3CA* mRNA overexpression are often high-grade carcinomas associated with myometrial invasion and tended to have lymphovascular invasion [15]. Furthermore, in high-grade endometrioid adenocarcinomas and mixed carcinomas, *PIK3CA* mutations in exon 20 coexist with *p53* alterations more frequently than in nonendometrioid adenocarcinomas. However, *PIK3CA* mRNA overexpression occurs in concert with *p53* alterations only in nonendometrioid endometrial carcinomas [15]. *PIK3CA* mutations did not correlate with MSI or *β*-catenin/*CTNNB1* mutations [9, 18]. *PIK3CA* mutations, particularly exon 20 mutations or *PIK3CA* mRNA overexpression, are frequent in endometrioid endometrial carcinoma in association with invasion and adverse prognostic factors such as blood vessel invasion [7, 15].

#### 2.3.3. KRAS

*KRAS* encodes a member protein of the small GTPase superfamily and is involved in signal transduction pathways between cell surface receptors and the nucleus. *KRAS* mutations have been identified in 10–30% of endometrioid endometrial carcinomas [1, 2, 4, 7–12, 17] while some investigators have reported an almost complete absence of *KRAS* mutations in serous and clear cell carcinomas of endometrium [8]. Some studies found a higher frequency of *KRAS* mutations in MSI-positive carcinomas than in MSI-negative tumors [8–10, 16] suggesting that both events may occur simultaneously before clonal expansion [10, 13]. *KRAS* mutations were detected in endometrial hyperplasias at a similar rate to that observed in endometrioid endometrial carcinomas, suggesting that *KRAS* mutations are early events in endometrial carcinogensis [9, 13]. No relationship has been found between *KRAS* mutations and tumor stage, histologic grade, depth of myometrial invasion, age, or clinical outcome in endometrioid endometrial carcinomas [9].

#### 2.3.4. *β*-Catenin (CTNNB1)

The *β*-catenin gene (*CTNNB1*) maps to 3p21. It appears to be important in the functional activities of both APC (adenomatous polyposis coli) and E-cadherin. It is a component of the E-cadherin-catenin unit, essential for cell differentiation and maintenance of normal tissue architecture and also plays an important role in Wnt signal transduction pathway. Mutations in exon 3 of *CTNNB1* result in stabilization of a protein that resists degradation, leading to nuclear accumulation of *β*-catenin, have been described in endometrioid endometrial carcinoma. The accumulation of *β*-catenin can be demonstrated by immunostaining. Several studies have analyzed endometrial carcinomas showing that nuclear accumulation of *β*-catenin is significantly more common in endometrioid lesions (31–47%) compared with nonendometrioid histology (0–3%) [4]. By comparison in colonic adenocarcinomas, elevated *β*-catenin levels caused by mutations in *CTNNB1* or APC result in activation of the Wnt/*β*-catenin/LEF1 pathway through a LEF1 binding site in the *cyclin D1* promotor, triggering *cyclin D1* gene expression, and subsequently, uncontrolled progression of tumor cells into the cell cycle [8, 12]. Furthermore, *β*-catenin might regulate the expression of the matrix metalloproteinase-7 that would have a role in the establishment of the microenvironment necessary for the initiation and maintenance of growth of the primary tumor and metastasis [8, 12]. The reported frequency of *CTNNB1* mutations in endometrioid endometrial carcinoma ranges from 14–44% [7, 8]. They seem to be independent from the presence of MSI and the mutations of *PTEN* and *KRAS*, suggesting that the Wnt pathway may play an independent role in endometrial cancer [10, 13]. In all cases, the mutations were homogeneously distributed in different areas of the tumors suggesting that they play a role in early steps of endometrial tumorigenesis. Alterations in *β*-catenin have been reported in endometrial hyperplasias with squamous metaplasia [7, 9]. Although there was a good correlation between *CTNNB1* mutations and *β*-catenin nuclear immunostaining, the presence of cytoplasmic and nuclear *β*-catenin immunoreactivity in some endometrial carcinomas without *CTNNB* mutation suggests that the changes of other genes in the Wnt/*β*-catenin/LEF-1 pathway may be responsible for the stabilization and putative transcription activator role of *β*-catenin [7, 8]. Endometrioid endometrial carcinomas with *CTNNB1* mutations are characteristically early stage tumors associated with favorable prognosis [7, 9]. Two members of the secreted frizzled-related protein (SFRP) family, SFRP1 and SFRP4, were more frequently down-regulated in MSI-positive carcinomas compared with MSI-negative carcinomas. This down-regulation was associated with frequent promoter methylation of SFRP1 and led to an activation of the *β*-catenin pathway. In addition, the Wnt-target fibroblast growth factor 18 was up-regulated in endometrioid carcinomas with MSI compared with normal endometrium [1]. 

#### 2.3.5. Microsatellite Instability

Microsatellite DNA sequences are polymorphic, short-tandem repeats distributed throughout the genome. The most common microsatellite in human is a dinucleotide repeat of CA, (CA)n, and there are 50,000 to 100,000 (CA)n repeats scattered in the human genome [8, 9]. Microsatellite instability (MSI) is a condition manifested by damaged DNA because of defects in normal DNA repair process. Mammalian mismatch repair (MMR) genes encode for nine proteins (MLH1, MLH3, PMS1, PMS2, MSH2, MSH3, MSH4, MSH5, and MSH6) that interact with each other to form complexes and heterodimers that mediate distinct functions in MMR-related system. This repair process plays a central role in promoting genetic stability by repairing DNA replication errors, inhibiting recombination between non-identical DNA sequences and participating in responses to DNA damage. MSI is a common genetic abnormality that has been detected in 20–45% of sporadic endometrioid endometrial carcinoma [7–10]. In addition, MSI in nonendometrioid endometrial carcinomas has been reported (0–11%) [8, 9], particularly in mixed endometrioid and serous carcinomas, but not in pure serous carcinomas [10]. In sporadic endometrial carcinoma, epigenetic cause of MSI is more common involving MLH1 promotor hypermethylation which is the main cause of MMR deficiency [7–9, 13, 15]. This epigenetic inactivation usually occurs in atypical hyperplasia, most of which coexists with carcinomas. Thus, MLH1 hypermethylation is an early event in the pathogenesis of endometrioid endometrial carcinoma, which precedes the development of MSI [7–9, 15]. The remaining unmethylated MLH1 cases reveal MSH2 mutations (15%) and MSH6 mutations (60%), of which almost half are germline mutations. Thus, MSH6 mutations seem to be a frequent cause of MSI [11, 12]. Tumors with MSI of CpG island methylation in the promoter region have been identified in some other genes, for example, *p16, PTEN*, and E-cadherin (*CDH1*), suggesting altered methylation may be a coexisting independent early change [9]. The presentation of some small short-tandem repeats such as mononucleotide repeats located within the coding sequence of important genes for example, transforming growth factor *β* receptor type II (*TGF-*β*RII*), *BAX*, insulin-like growth factor II receptor (*IGFIIR*), *MSH3, MSH6, caspase-5*, and *PTEN* may promote MSI-positive endometrial carcinoma [8, 9]. Secondary mutations at one or more mononucleotide tracts found in 72.7% of tumors with MSI, are responsible for tumor progression [7–9]. International Federation of Gynecology and Obstetrics (FIGO) grade has been found to be higher in endometrioid endometrial carcinomas with MSI in some, but not all studies, similar to the well-established association between MSI and high-grade colorectal carcinomas [16]. By multivariate analysis, a significant correlation between MSI-positive tumors and tumor-infiltrating lymphocytes in endometrioid endometrial carcinoma was found: 40 tumor-infiltrating lymphocytes/10 high power fields has a sensitivity of 85% and a specificity of 46% in predicting MSI [16]. 

### 2.4. Molecular Pathology of Nonendometrioid Carcinomas

#### 2.4.1. p53

The *p53* tumor suppressor gene locates to chromosome 17p13.1. While *p53* mutations occur in 90% of non-endometrioid endometrial carcinoma, they are only present in 10–20% of endometrioid endometrial carcinoma, which are mostly high-grade [7, 18]. The abnormal p53 expression has been found in 11% of grade 1 endometrioid endometrial carcinoma [18]. This finding supports that *p53* mutations may influence progression of endometrioid endometrial carcinomas to non-endometrioid endometrial carcinomas [9]. In fact, *p53* mutation is the most characteristic genetic alteration of non-endometrioid endometrial carcinomas [9, 10] and may be useful in their distinction from endometrioid endometrial carcinomas [22]. In *p53* positive endometrioid endometrial carcinoma, p53 protein accumulation may be secondary to changes in its upstream regulatory proteins rather than the *p53* gene itself. Several genes, including *MDM2* and *p14 AR*, that regulate *p53* levels have been shown to cause detectable levels of *p53* in the absence of *p53* mutation. Alternatively, nonspecific DNA damage such as that induced by irradiation is also known to induce accumulation of wild-type *p53* [12]. In normal cells, *p53* is rapidly degraded and thus cannot be detected by immunostaining. *p53* mutations produce a non-functional protein that resists degradation and can be visualized by immunostaining [11, 18]. However, loss of function of *p53* resulting from LOH may not correlate with protein overexpression. In addition, frameshift mutations and stop codons lead to a truncated protein, which is not detected by antibodies and leads to negative immunohistochemistry [11, 18] After DNA damage, nuclear *p53* accumulates and causes cell cycle arrest by inhibiting *cyclin D1* phosphorylation of the *Rb* gene and thereby promoting apoptosis [9, 13]. Overexpression of *p53* is associated with high histological grade and advanced stage as well as unfavorable prognosis [9, 18]. Endometrial intraepithelial carcinoma (EIC), the putative precursor lesion to serous carcinomas [4, 13, 18, 22, 23], characterized by replacement of the surface epithelium by malignant cells exhibiting cytological features similar to those of serous carcinoma [9, 23]. EIC has been reported in nearly 90% of uteri containing serous carcinoma that is often extensive and multifocal [23]. Mutations of *p53* are also found in 75–80% of EIC. It is postulated that mutation in one allele occurs early during the development of serous carcinoma, and loss of the second normal allele occurs late in the progression to carcinoma [4]. *p53* mutations are almost always associated with aneuploidy and do not seem to occur with *PTEN* mutations in the same tumor [10, 11].

#### 2.4.2. HER2/neu

Epidermal growth factor receptor II or *HER2/neu* is an oncogene that codes for a transmembrane receptor tyrosine kinase involved in cell signaling and located at the long arm of human chromosome 17q12. *HER2/neu* overexpression or amplification is more frequently found in non-endometrioid endometrial carcinoma (18–80%) [13] than in grade 2 and 3 endometrioid carcinoma (10–30%) [7, 9, 10] and has been associated with adverse prognostic parameters including advanced stage, high histologic grade, and low overall survival [9, 13].

#### 2.4.3. p16

*p16* plays an important role in regulating the cell cycle. It is a tumor suppressor gene located on chromosome 9p21 [10]. *p16* inactivation can lead to uncontrolled cell growth. Inactivation of *p16* is more frequent in non-endometrioid endometrial carcinoma (40–45%) than in endometrioid endometrial carcinoma (10%) [4, 7, 10]. The underlying mechanism is unclear [7, 11], because neither promoter hypermethylation nor deletion or mutation is frequently found [11]. Loss of *p16* expression is correlated with *KRAS* and *p53* mutations and is associated with high stage, high grade, and poor survival [10].

#### 2.4.4. E-Cadherin

Cadherins are a family of adhesion molecules essential for tight connection between cells. E-cadherin is encoded by *CDH1* gene and locates on chromosome 16q22.1. It is thought to be a tumor suppressor gene, the loss of which has been demonstrated to promote tumor invasion and metastasis. Decreased expression of E-cadherin is frequent in endometrial carcinoma and may be caused by LOH or promotor hypermethylation. LOH at 16q22.1 is seen in almost 60% of non-endometrioid endometrial carcinoma, but in only 22% of endometrioid endometrial carcinoma [7]. In endometrial carcinoma, partial or complete loss of E-cadherin expression correlates with aggressive behavior [9].

Among type II carcinomas, clear cell carcinomas seem to follow a separate pathway that shows some overlap with serous and endometrioid carcinomas. *p53* mutations are only present in about 30–40% of clear cell carcinomas compared to 90% of serous carcinomas. However, the frequency of MSI and *PTEN* alterations in clear cell carcinoma is higher than in serous carcinoma (15% versus <5 for MSI and 30% versus 10% for *PTEN*) but lower compared with endometrioid carcinoma (20–40% and 35–50%, resp.) [24]. A recent molecular study demonstrated that the majority of pure clear cell carcinomas do not show mutations in either *PTEN* or *p53*, the most commonly altered genes in type I and type II tumors, respectively. These findings suggest that clear cell carcinoma may arise through a distinct pathologic pathway [6]. 

#### 2.4.5. Apoptosis Resistance in Endometrial Carcinoma

Several of the molecular abnormalities that have been detected in EC may be associated with apoptosis deregulation. Apoptosis can be initiated by two main mechanisms: (i) the “intrinsic pathway” activated by released mitochondrial proteins, such as cytochrome-c; and (ii) the “extrinsic pathway” activated by ligand-bound death receptors such as tumor necrosis factor (TNF), Fas or TNF-related apoptosis including ligand (TRAIL) receptors. Some studies have shown that cellular apoptosis susceptibility (*CAS*) gene, *BCL2, BAX*, and caspase-3 are apparently involved in the progressive deregulation of proliferation and apoptosis, leading from simple and complex endometrial hyperplasia to adenocarcinoma. As described above, *PTEN* antagonizes the PI3K/AKT pathway by dephosphorylating PIP3, resulting in decreased translocation of AKT activation. Thus, loss of *PTEN* function leads to increased levels of phospho-AKT, activation of anti-apoptotic protein, and cell cycle progression [9]. NF-*κ*B, frequently activated in endometrioid endometrial carcinomas, may inhibit apoptosis by activation of target genes such as *FLIP* and *Bcl-XL*. Furthermore, there are reports that apoptosis-related protein survivin is frequently overexpressed in endometrial carcinomas [7, 9] and correlates inversely with *PTEN* expression [9]. Where widespread genetic abnormalities exist that cannot be corrected, MMR proteins initiate apoptosis as a more energy efficient option of universal genomic preservation [16]. MMR deficiency lowers the apoptotic rate, leading a survival advantage to the mutated cells [16].

#### 2.4.6. cDNA Array Studies

cDNA analyses have demonstrated that the expression profiling of endometrioid endometrial carcinoma is different from that of non-endometrioid endometrial carcinoma. These studies have identified gene signatures specific for non-endometrioid endometrial carcinomas as well as genes specifically up- or down-regulated in endometrioid endometrial carcinomas when compared with normal endometrium. Intestinal trefoil protein, *TFF3, AGR2* developmental gene, estrogen-regulated genes (*MGB2, LTF, END1, MMP11*), *FOXA2*, and *MSX2* were significantly up-regulated in endometrioid endometrial carcinomas, while increased expression of *FOLR*, genes involved in the regulation of mitotic spindle checkpoint (*STK15, BUB1, CCNB2*), *IGF2, PTGS1* and *p16* were seen in non-endometrioid endometrial carcinomas. *STK-15* also known as *BTAK, *Aurora-A, is a serine/threonine kinase which is essential for chromosome segregation and centrosome functions [7, 9]. Overexpression of *STK15* induces increased numbers of centrosomes, aneuploidy, and malignant transformation. One study found *STK15* amplification in 9 of 15 (60%) non-endometrioid endometrial carcinomas but in none of endometrioid endometrial carcinomas [9]. Furthermore, a different expression profile was also found between endometrial carcinoma associated with MSI and stable endometrial carcinoma. *SFRP1* and *SFRP4* were more frequently down-regulated in endometrial carcinoma with MSI. One study compared the expression profiles of similar histological subtypes of ovarian and endometrial carcinomas, and showed that clear cell carcinomas had a very similar profile, regardless of the organ of origin. In contrast, differences were seen when comparing endometrioid and serous carcinomas of ovarian and endometrial origin [7].

## 3. Genetic Changes in Endometrial Carcinogenesis (Progression Models) of Endometrioid and Serous Carcinomas, Including Molecular Changes of Premalignant Disease (Hyperplasia/EIC)

By epidemiological and molecular evidence, endometrial hyperplasia represents a true precursor lesion for endometrioid endometrial carcinomas, whereas non-endometrioid endometrial carcinomas are frequently associated with endometrial intraepithelial carcinoma (EIC) [13].

### 3.1. Progression Model for Endometrioid (Type I) Carcinomas

A progression model of endometrioid carcinoma resembling the Vogelstein progression model for colorectal carcinoma has been proposed. This hypothesis is supported by the evidence that (i) some of the genetic alterations found in endometrioid endometrial carcinomas are already present in atypical hyperplasia, (ii) increased genetic alterations are found in well-differentiated endometrioid carcinoma compared with atypical hyperplasia, (iii) the number of genetic alterations increase according to higher histologic grade, and (iv) more chromosomal imbalances are identified in endometrial carcinoma compared with atypical hyperplasia, using comparative genomic hybridization (CGH) [11]. 

**Figure 2:**
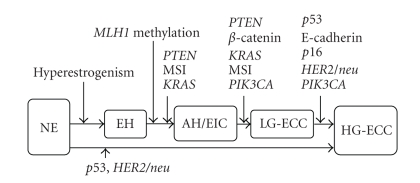
A progression model for endometrioid carcinoma. Tumor initiation and progression are characterized by acquisition of various molecular alterations. *PTEN* alterations appear central to the initiation of proliferative lesions that then acquire mutations in other cancer-causing genes (e.g., DNA mismatch repair genes, *KRAS*, *β*-catenin) in the carcinogensis. An alternative pathway bypasses atypical hyperplasia and low-grade carcinoma to high-grade carcinoma by *p53* mutation and *HER2/neu* amplification. NE, normal endometrium; EH, endometrial hyperplasia without hyperplasia, AH, atypical endometrial hyperplasia; EIC, endometrial intraepithelial carcinoma; LG-ECC, low grade endometrioid endometrial carcinoma; HG-ECC, high grade endometrioid endometrial carcinoma.

Most simple hyperplasias and a subset of complex hyperplasias are polyclonal and considered reactive processes due to hyperestrogenism, which may regress through progesterone therapy [11, 23]. In contrast, most atypical hyperplasias are monoclonal. A subset of complex hyperplasia without atypia has been reported to be monoclonal. In addition, the number of chromosomal aberrations in complex hyperplasia is significantly higher than simple hyperplasia and close to the number found in atypical hyperplasia. Most of the genetic alterations identified in endometrioid endometrial carcinoma seem to occur very early in the development of endometrioid carcinoma, although it is not clear which alterations are associated with the earliest changes of malignant transformation and progression to neoplasia [10, 11]. In atypical hyperplasia, alterations of *PTEN*, *β*-catenin, *KRAS*, and MSI are present, with *PTEN* inactivation occuring in about 50% of the cases. However, *PTEN* and *KRAS* mutations seem to occur earlier, since they were found in simple hyperplasia, partially associated with monoclonality. *PTEN* inactivation has been reported in normal endometrial glands but its significance is yet unknown [11]. The inactivation of E-cadherin gene by methylation seems to play a role during progression of endometrioid carcinoma, since it is most frequently found in grade 3 and least frequently in grade 1 tumors [10]. Furthermore, *p53* mutations, *HER2/neu* overexpression or amplification, and *p16* inactivation are considered in late events during carcinogenesis of endometrioid carcinoma, since they are predominantly identified in grade 3 tumors, but rarely in grade 1 tumors, and are absent in atypical hyperplastic lesions. Hypothetically, *p53* mutations and *HER2/neu* amplification might also be early events in *de novo* poorly differentiated endometrioid carcinomas [10, 11] (Figure 2). Endometrial pre-cancers (e.g., EIC) have been postulated to share common genetic alterations with endometrioid endometrial carcinoma, including *PTEN* mutations and MSI [13].

### 3.2. Progression Model for Nonendometrioid (Type II) Carcinomas

**Figure 3:**
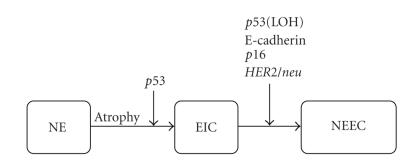
A progression model for nonendometrioid (type II) carcinomas. *p53* mutations play a critical role in the conversion of atrophic endometrium to an intraepithelial form of serous carcinoma. NE, normal endometrium; EIC, endometrial intraepithelial carcinoma; NEEC, non-endometrioid endometrial carcinoma.

Mutations of *p53* were found in approximately 80% of EIC, but in contrast to most serous carcinomas, there is no LOH at the locus TP53. Thus, it is hypothesized that *p53* mutation of one allele occurs early, whereas loss of the normal second allele accompanies progression into serous carcinoma [10, 11]. The alterations of E-cadherin, *p16*, and *HER2/neu* seem to affect the progression from EIC to serous carcinoma [10]. Another group hypothesized that serous carcinoma may develop from endometrioid carcinoma through *p53* mutation based on findings in mixed endometrioid and serous carcinomas. Early genetic alterations during carcinogenesis are not clear, as these authors presented no data for EIC [10, 11] (Figure 3).

## 4. Hereditary Endometrial Carcinoma

Hereditary endometrial carcinoma has been found in 2–5% of endometrial cancer [24]. Hereditary nonpolyposis colon cancer (HNPCC), also known as Lynch syndrome or cancer family syndrome, accounts for the majority of inherited cases [24]. It is an autosomal dominant syndrome that predisposes its carriers to multiple malignancies particularly colorectal, and endometrial carcinomas [25], caused by a germline mutation in one of the DNA MMR genes occurring in 30–60% of cases [8, 9]. Endometrial carcinoma is the most common extracolonic malignancy in patients with HNPCC. In women with HNPCC, the incidence of endometrial carcinoma equals or exceeds that of colorectal carcinoma, compared with 1% in the general population [26], and in more than 50% of HNPCC cases, these women present with a gynecological cancer as their first or “sentinel” malignancy [25]. The frequency of germline DNA MMR gene mutations among unselected patients with endometrial carcinoma has been found to be 1.8–2.1%, which is similar to the frequency of HNPCC in colorectal carcinoma [25]. Patients with endometrial carcinoma in the HNPCC population have an inherited germline mutation in *MLH1, MSH2, MSH6*, or *PMS2* (first hit) but endometrial carcinoma develops only after the initiation of a deletion or mutation in the contralateral *MLH1, MSH2, MSH6*, or *PMS2* allele (second hit) in endometrial cells [7, 8]. Once the 2 hits have occurred, the deficient MMR function of *MLH1, MSH2, MSH6*, or *PMS2* causes the acquisition of MSI and subsequent tumor development [7–9]. Unlike HNPCC associated colorectal carcinoma, which appears to frequently have *MLH1* and *MSH2* mutations, endometrial carcinomas have a higher probability of *MSH2* and *MSH6* mutations [13, 25]. Women with HNPCC who carry *MSH2* and *MSH6* mutations have a higher chance to present initially with endometrial rather than colorectal cancer [16]. MSI has been detected in 75% of endometrial carcinoma associated with HNPCC [8, 9]. Many studies have shown that MSI is associated with endometrioid histologic type. However, 14–21% of HNPCC-associated endometrial carcinomas are non-endometrioid, but only 3.3–4.5% of sporadic MSI tumor [16]. Women with an inherited predisposition for endometrial neoplasia tend to develop the disease 10 years earlier than the general population [9]. There is 18–23% incidence of HNPCC syndrome in endometrial carcinoma patients younger than 50 years old [16]. In addition to endometrial carcinoma arising from HNPCC, occasional families show clustering of endometrial cancer alone, without colon or other cancers. This group was termed as “familial site-specific endometrial cancer” [10]. Loss of protein expression seems to occur frequently for both *MLH1* and *MSH2* in endometrial hyperplasia and is considered an early event during tumor development [11]. *PTEN* inactivation by mutation seems to also be involved in tumorigenesis, since it occurs in about 90% of type I carcinomas [11]. Currently, there are no data to suggest that the prognosis for women with HNPCC-associated endometrial cancers is either better or worse than for women with sporadic cancers [16, 24]. In one study, endometrial carcinoma in HNPCC kindreds was a cause of death in 12% of cases; in 61% of cases these patients had a second primary malignancy; and 15% of cases had more than 2 additional primary cancers. Nieminen et al. [26] studied serial endometrial biopsy samples taken during a 10-year followup of HPNCC mutation carriers and found abnormal MMR protein expression, MSI, or tumor suppressor promotor hypermethylation in various endometrial histologies, including normal and hyperplastic endometria. The most frequently methylated genes were *CDH13, RASSF1A*, and *GSTP1*. These defects in MMR and methylation appeared up to 12 years before endometrial carcinoma [26].

PTEN hamartoma tumor syndrome, caused by a germline mutation in *PTEN* gene on chromosome 10q, comprises a group of disorders including Cowden syndrome, Bannayan-Riley-Ruvalcaba syndrome, Proteus syndrome, Proteus-like syndrome, and autism spectrum disorder with macrocephaly [27, 28]. Cowden syndrome, also known as multiple hamartoma syndrome, is an autosomal dominant disorder with high risk of breast, thyroid, and endometrium cancer. The incidence of Cowden syndrome remains unclear due to underdiagnosis from variable penetration and subtle clinical findings [29]. Cowden syndrome is characterized by the development of intestinal hamartomas, facial trichilemmomas and mucocutaneous papillomatosis [29, 30], and is rarely identified before adulthood [28]. *PTEN* mutations in exon 5, coding for the active site and flanking amino acids, is a common site for mutations in patients with Cowden syndrome, and missense mutations are only found in this active area [30]. However, germline *PTEN* mutation has been detected in approximately 80% of Cowden syndrome patients [27]. The lifetime risk for endometrial carcinoma in Cowden syndrome is approximately 5–10%, compared with a 2.5% lifetime risk of women in the general population [27, 28]. Cowden syndrome and Bannayan-Riley-Ruvalcaba syndrome have overlapping phenotypic features. Bannayan-Riley-Ruvalcaba syndrome is a congenital, autosomal dominant condition manifested by macrocephaly, hamartomatous intestinal polyposis, lipomas, developmental delay or autism or both, and pigmented penile macules [29, 31]. Unlike Cowden syndrome, Bannayan-Riley-Ruvalcaba syndrome tends to be diagnosed at early life [28]. Approximately 60% of Bannayan-Riley-Ruvalcaba syndrome patients have an identifiable germline mutation in *PTEN* gene [27]. This syndrome also has the same increased risk of cancer as Cowden syndrome [29].

## 5. Carcinosarcoma

**Figure 4:**
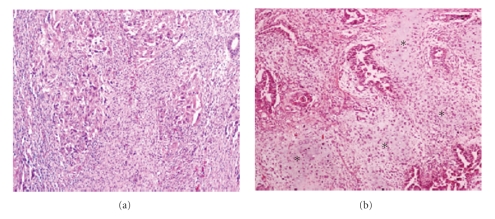
Carcinosarcoma is composed of two malignant components, carcinomatous and sarcomatous. The epithelial component is usually high grade carcinoma for example, serous/clear cell type. The mesenchymal part comprises either homologous (a), ×100 or heterologous element for example, cartilage or bone. Chondrosarcomatous element (∗) is present in (b), ×100.

Carcinosarcomas (formerly known as malignant mixed mesodermal or mullerian tumors) are highly aggressive, biphasic neoplasms composed of carcinomatous and sarcomatous components (Figure 4). Carcinosarcomas account for 1-2% of all malignancies of uterine corpus [1] and usually present in postmenopausal women. Uterine bleeding is the most frequent presenting symptom. These tumors have traditionally been regarded as a subtype of uterine sarcomas or as a mixture of true carcinoma and sarcoma, but they are now regarded as metaplastic carcinomas or carcinomas with sarcomatous metaplasia [1, 18, 32–34]. Carcinosarcomas can be classified as type II endometrial carcinomas and their epithelial component can resemble high grade endometrioid, serous or clear cell carcinoma [35]. Etiologically, a few cases may be secondary to prior pelvic irradiation. In addition, an association between long term tamoxifen therapy and development of uterine carcinosarcoma has been suggested [32].

Schipf et al. [36] analyzed a series of 30 paraffin-embedded carcinosarcomas, including 24 ovarian and 6 uterine, using fluorescence in situ hybridization (FISH) and CGH. Many carcinosarcomas contained aberrations on chromosome 8 and 20 detected by FISH. FISH showed *C-MYC *(8q24.12) and *ZNF217* (20q13.2) amplification in 78% and 87%, respectively. The results demonstrate a uniform pattern of chromosomal gains and losses in CGH analysis. Gains or amplifications of 8q are the most common genetic aberration in carcinosarcomas [35]. One of the genes located within 8q is *C-MYC* (8q24) found to be amplified in 18 of 23 uterine and ovarian carcinosarcomas through FISH and overexpression in 9 of 9 uterine carcinosarcomas through immunostaining. *C-MYC* amplification is often present in carcinomas but was also present in 6 of 12 uterine leiomyomas and 11 of 23 uterine leiomyosarcomas [35]. 

LOH was seen in 5 of 6 uterine carcinosarcomas, and identical alleles were lost in the epithelial and mesenchymal components. *p53* mutations and LOH for TP53 occur frequently in both carcinosarcoma components which are associated with frequent protein overexpression. Sherman et al. [18] reported immunoreactivity of p53 in 7 (70%) of 10 carcinosarcomas and noted that the similar staining pattern presented in both carinomatous and sarcomatous areas. In about 20% of carcinosarcomas, MSI-high was described with an 83% concordance between the carcinomatous and sarcomatous components [1]. One study found identical mutations of *p53* and *KRAS* in the two components [33]. Fujii et al. [37] analyzed allelic status with polymorphous microsatellite markers on 172 carcinomatous/sarcomatous foci after microdissection of 17 carcinosarcomas. A close relationship between the carcinomatous and the sarcomatous component was found. No difference was seen in CGH patterns of carcinosarcomas [36]. Moreover, there is evidence that in most carcinosarcomas, the carcinomatous and the sarcomatous components are genetically the same, as shown for 21 of 25 carcinosarcomas (84%) using the human androgen receptor (HUMARA) for detection of X-chromosome inactivation. These results support a monoclonal origin of uterine carcinosarcomas and one can hypothesize that either the sarcomatous component develops from the carcinomatous component (conversion theory) or both are derived from a stem cell that undergoes divergent differentiation (combination theory) [33]. In the process of epithelial-mesenchymal transition, cells of epithelial origin lose epithelial characteristics and polarity acquiring a mesenchymal phenotype with increased migratory behavior. By molecular mechanisms, down-regulation of epithelial markers and up-regulation of mesenchymal markers result in acquisition of a fibroblast-like morphology with cytoskeleton reorganization and increase in motility, invasiveness, and metastatic capacity. A hallmark of epithelial-mesenchymal transition is loss of E-cadherin expression. A number of specific transcription factors, including Snail, Slug, SIP-1, and Twist, contribute to induction of epithelial mesenchymal transition, acting as transcriptional repressors of the E-cadherin gene. The oncogenic serine/threonine kinase AKT also promotes the process, modulating several signaling and transcriptional networks linking Wnt/*β*-catenin, NF-*κ*B/p65, and Rb [38]. However, some investigators also found that a subset of carcinosarcoma was biclonal tumor, consisting of independent unrelated carcinomas and sarcomas, according to X chromosome inactivation and clinicopathological criteria [35]. In two collision reported tumors, the carcinomatous and sarcomatous components were histologically separate, with no intermingling, and there was a nodal metastasis that consisted purely of the sarcomatous element from one of these tumors [33]. One study examined 26 carcinosarcomas and found adenosarcoma-like components in 4 cases, suggesting that many of the true collision lesions may arise from malignant transformation of either benign epithelium within an adenosarcoma or adjacent benign endometrium [35]. The prognosis of the collision tumor depends on the most aggressive component, and may be better than for a carcinosarcoma of similar stage [33]. Overall, the carcinomatous component has been shown to have a more aggressive behavior and be a better predictor of clinical outcome in carcinosarcomas [35].

## 6. Endometrial Stromal Sarcoma and Undifferentiated Endometrial Sarcoma

**Figure 5:**
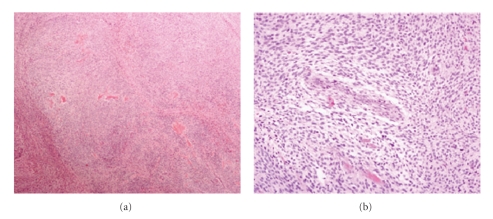
Endometrial stromal sarcoma, low grade is circumscribed from the surrounding myometrium (a), ×40; and higher magnification of endometrial stromal sarcoma shows round uniform tumor cells resembling the stroma of proliferative endometrium with low mitotic rate (b), ×200.

Endometrial stromal sarcoma and undifferentiated endometrial sarcoma are in the same neoplastic spectrum. Diagnosis of endometrial stromal tumors has been based on histologic criteria. Low grade endometrial stromal sarcoma is composed of uniform, oval to spindle-shape cells of endometrial stromal-type with numerous small arterioles that resemble the spiral arterioles of late secretory endometrium. Mitotic rate is not a consideration in the distinction between low and high grade stromal sarcoma. In addition, characteristic tongue-like growth of the stromal cells into the myometrium and/or myometrial vasculature is noted [39, 40] (Figure 5). Endometrial stromal sarcoma usually occurs in middle aged women [41], and most present with uterine bleeding. Undifferentiated endometrial sarcoma, on the other hand, is defined as a high-grade neoplasm that lacks specific differentiation and bears no histological resemblance to endometrial stroma. Also, undifferentiated endometrial sarcomas have marked nuclear pleomorphism with high mitotic rate and display destructive myometrial invasion [40, 42]. Undifferentiated endometrial sarcomas should be diagnosed only after extensive sampling to exclude smooth or skeletal muscle differentiation, to exclude high grade leiomyosarcoma or rhabdomyosarcoma. Carcinosarcoma or adenosarcoma with sarcomatous overgrowth should also be excluded before making the diagnosis of undifferentiated endometrial sarcomas [39, 40]. 

In endometrial stromal sarcomas, the tumor cells are typically immunoreactive for estrogen and progesterone receptors, CD10, vimentin, and sometimes focally with actin, while they are generally negative for desmin, and h-caldesmon. Expression of androgen receptor is observed in 41% of examined sarcoma cases [41]. Approximately 70% of low grade endometrial stromal sarcomas also expresses epidermal growth factor receptor (EGFR; HER1). Undifferentiated endometrial sarcomas are estrogen and progesterone receptor negative, but a high proportion is EGFR positive. Endometrial stromal sarocmas are typically diploid with a low S-phase fraction whereas S-phase fraction exceeds 10% in undifferentiated endometrial sarcomas [41]. No c-kit (CD117) expression has been demonstrated in endometrial stromal sarcomas [43]. Liegl et al. [43] found 22 of 37 endometrial stromal sarcomas showed platelet-derive growth factor (PDGF)-*α* (CD140*α*) and 8 of 37 endometrial stromal sarcomas showed PDGF-*β* expression.

In contrast to epithelial endometrial carcinoma, endometrial stromal tumors are characterized by distinct cytogenetic abnormalities, particularly translocations leading to gene fusion. Cytogenetic studies reported to-date are primarily for low grade endometrial stromal sarcomas, mostly showing rearrangement of chromosomes 6, 7, and 17 [44, 45]. Loss of chromosome arm 7p (55.6% of the cases) is the most frequent aberration and may play a role in tumor development and progression [41]. Reverse transcription polymerase chain reaction (RT-PCR) and FISH studies on large series showed the presence of t(7;17)(p15;q21), leading to the fusion of two zinc finger genes, *JAZF1* (juxtaposed with another zinc finger gene 1) and *JJAZ1* (joined to *JAZF1*; also named *SUZ12*, suppressor of zeste-12 protein). *JAZF1* is expressed in normal endometrial stroma, but the specific functions of the *JAZF1* and the *JJAZ1* genes as well as the *JAZF1/JJAZ1* fusion gene are still unknown [1, 42]. Based on the evidence of loss of expression for normal versions of *JAZF1* in multiple tumors suggests a possible role of this gene as a tumor suppressor [42]. This gene fusion is a distinctive molecular genetic alteration for endometrial stromal sarcoma and benign endometrial stromal nodules [1, 41]. The *JAZF1/JJAZ1* fusion gene is frequently present in classical endometrial stromal sarcomas and less often in cases with variant histology [46]. However, of seven high-grade endometrial stromal sarcomas/undifferentiated endometrial sarcomas studied, only three cases showed evidence of the fusion [42]. In contrast, many studies reported the fusion gene to be absent in undifferentiated endometrial sarcomas [41]. The fusion gene is not present in normal endometrial stroma [41]. The presence of the *JAZF1/JJAZ1* fusion gene within the spectrum of endometrial stromal tumors from benign to malignant raises possibility that the endometrial stromal nodule may transform into malignant endometrial stromal sarcoma [41, 47]. The frequencies of this gene fusion in low grade endometrial stromal sarcoma have been reported in many studies showing a wide range of positivity, 23–80% [44, 46, 48, 49]. The studies with RT-PCR only can give false-positive results due to PCR contamination. FISH may be useful as a complementary technique to exclude the possibility of false positive contamination of cases by RT-PCR [44]. Although the *JAZF1/JJAZ1* fusion gene seems to be the major molecular alterations in endometrial stromal sarcomas, there is some evidence for alternative pathways in the development of endometrial stromal sarcomas. A major subgroup of endometrial stromal sarcomas has been found to have translocations involving short arm of chromosome 6, particularly band 6p21 [41, 44, 45]. Micci et al. [50] showed that the *PHF1* (PHD finger protein 1) gene in 6p21 was recombined with two different partners, (i) with *JAZF1* gene showing a 6p;7p rearrangement, which results in the formation of a *JAZF1/PHF1* fusion gene and (ii) with *EPC1* (enhancer of polycomb) gene in 10p11 that had a 6;10;10 translocation. Panagopoulos et al. [45] introduced that a low-grade endometrial stromal sarcoma cell line carrying a der(7)t(6;7)(p21;p22) also harbors a *JAZF1/PHF1* fusion. Both t(7;17) and t(6;7) comprise 62% of the reported endometrial stromal sarcomas [44]. Additionally, few endometrial stromal sarcoma cases were reported with a t(X;17)(p11.2;q23) and a t(10;17)(q22;p13) [51–53]. Although *JAZF1/JJAZ1* fusion may not be universally present in all low grade endometrial stromal sarcoma, this aberration may still be diagnostically useful [44]. The *JAZF1/JJAZ1* fusion has been identified in areas of smooth muscle differentiation in endometrial stromal neoplasms (50% of the cases). This finding supports that the endometrial stromal and smooth muscle components of these tumors have the same origin, either from a common precursor cell with pluripotential differentiation or from endometrial stromal cells that have undergone smooth muscle metaplasia [44, 54]. Halbwedl et al. [55] described 9 cases of low grade endometrial stromal sarcoma and 3 cases of undifferentiated endometrial sarcoma in aCGH study revealing a variety of gains and losses that apparently did not correlate with morphology. There is no accumulation of aberrations in undifferentiated endometrial sarcoma compared to endometrial stromal sarcoma, indicating these two types of uterine sarcomas are probably not related to each other. 

LOH and MSI have been evaluated in both low grade endometrial stromal sarcomas (20 cases) and undifferentiated endometrial sarcomas (3 cases). LOH with at least one polymorphic DNA marker was identified in all 3 cases (100%) of undifferentiated endometrial sarcomas, 10 (50%) low-grade endometrial stromal sarcomas and 2 (50%) benign endometrial stromal nodules. Moreover, concurrent and independent LOH were noted in adjacent normal appearing myometrium or endometrium, either close to or at a distance from the tumors [41]. LOH was mostly identified at *PTEN*, a tumor suppressor gene located on chromosome 10q [56]. No tumor was associated with MSI [41, 55]. Loss of functions of certain tumor suppressor genes such as *PTEN* in surrounding nontumor uterine tissues could influence and facilitate tumor proliferation, cellular spread, and invasion by malignant endometrial stromal cells [41]. However, one should keep in mind the false positive scoring of LOH in normal tissues may occur both from the imperfect methodology and from contamination by tumor samples/cells. The use of repeated experiments and several polymorphic markers has been advised to overcome these methodology problems [56]. Other frequently altered loci by LOH were at 14q32 (D14S267) and 3p (D3S1300). The former locus is frequently altered in uterine leiomyosarcoma but, in addition, in a variety of epithelial neoplasms such as ovarian, colorectal and esophageal carcinoma. Locus D3S1300 harbors the *FHIT* gene which is frequently mutated in cervical carcinoma of the uterus. LOH for TP53 and p53 overexpression are rarely present in endometrial stromal sarcomas (5% and 15%, resp.). The importance of *p53* mutations for the development of undifferentiated endometrial sarcomas is not evident, but p53 overexpression was detected in three of four high-grade stromal sarcomas [1]. Furthermore, Kurihara et al. [49] have recently found frequent nuclear accumulation of p53 and *TP53* gene missense mutations in undifferentiated endometrial sarcoma with nuclear pleomorphism, 3 (50%) of 6 cases. There is no evidence of *p53* aberration in 18 low grade endometrial stromal sarcomas and 7 cases undifferentiated endometrial sarcoma with nuclear uniformity. *p53* alteration may be one different pathway that contributes the tumorigenesis of undifferentiated endometrial sarcoma. Expression of *SFRP4* and *β*-catenin is also detected. *SFRP4* acts in Wnt-signaling pathway, which is a complex cascade of heterogeneous molecules playing an important role in organ development, via *β*-catenin. *SFRP4* is expressed in normal endometrial stromal cells but not in glandular epithelium. Compared with normal endometrium, the expression of *SFRP4* was decreased in both low grade endometrial stromal sarcomas and undifferentiated endometrial sarcomas. Through its involvement in the Wnt signaling pathway, *SFRP4* may act as a tumor suppressor by regulating the cytosolic *β*-catenin pool in the cell. Beta-catenin regulates in the opposite manner to *SFRP4*, being particularly increased in undifferentiated sarcoma [57]. Dysregulation of these pathways allows *β*-catenin to accumulate and translocate to the nucleus, where it forms complexes with T-cell factor/lymphoid enhancing factor (TCF/LEF) leading to uncontrolled cell growth and carcinogenesis [57]. High level nuclear staining for *β*-catenin was seen in 40% of endometrial stromal sarcomas and may be used as a diagnostic tool [42].

## 7. Diagnostic Utility Based on the Molecular Knowledge

### 7.1. Endometrioid Carcinoma versus Serous/Clear Cell Carcinoma

At times, the histological type of endometrial carcinoma is not clearly defined, especially in poorly differentiated tumors, and knowledge of the dualistic model, with the common molecular changes in each type, can help clarify the diagnosis. If there is non-carcinomatous endometrium present, the presence of hyperplasia is supportive evidence of an endometrioid carcinoma, whereas atrophic endometrium is supportive of non-endometrioid carcinoma. 

Molecular studies on endometrium are not often performed in most hospital surgical pathology laboratories today; however, immunohistochemical studies can detect the abnormal protein products of the gene mutations. Therefore, we can exploit our knowledge of the dualistic model and their typical gene mutations and use the immunoprofile as a diagnostic tool, in concert with the histomorphologic features to specify the tumor type, particularly in difficult cases such as in the differentiation between high-grade endometrioid carcinoma and serous carcinoma (Table 3).

### 7.2. Endometrial Stromal Sarcoma versus Undifferentiated Sarcoma

The distinct molecular alteration described in the majority of endometrial stromal sarcomas is the t(7;17)(p15;q21) leading to the formation of fusion gene *JAZF1/JJAZ1*, which can be detected by RT-PCR or FISH assays. Thus, in the problematic cases in which the differentiatial diagnosis is between endometrial stromal sarcoma and undifferentiated sarcoma, we look for the fusion gene to make this distinction.

### 7.3. Uterine Smooth Muscle Neoplasm versus Endometrial Stromal Tumors

Uterine smooth muscle neoplasm is defined as a mesenchymal tumor composed of cells with smooth muscle differentiation, particularly highly cellular leiomyomas may have morphologically overlapped features of endometrial stromal tumors. According to histologic criteria for differential, immunostainings may help to correct the final diagnosis, particularly in difficult cases. Neoplastic endometrial stromal cells typically express vimentin, muscle-specific and smooth muscle actin and may be positive for desmin. In addition, CD10, initially thought to be a specific marker for endometrial stromal tumors, can be demonstrated in uterine smooth muscle tumors, commonly in highly cellular leiomyomas and leiomyosarcomas. Other antibodies that give positive staining in smooth muscle tumors useful in this differential diagnosis includes h-caldesmon, histone deacetylase 8 (HDAC8), smooth muscle myosin and oxytocin receptor [39]. However, none of these markers can completely specify the smooth muscle/endometrial stroma lineage of the tumor, a panel of the antibodies should be used [39]. 

The molecular alterations in smooth muscle tumor are complex, especially in leiomyosarcomas. The translocation t(12;14)(q15;q23-24) has been noted in a high proportion of leiomyomas [41, 58]. By CGH, leiomyosarcomas have the most frequent losses including 10q, 11q, 13q, and 2p while the most common gains are Xp, 1q, 5p, 8q, 12q, 17p and 19p [41, 59, 60]. There are a variety of genetic changes and mutations inclusive of *TP53* and *MDM2* expression associated with progression of leiomyosarcomas [41]. LOH of 10q is found in more than half of leiomyosarcomas [41]. Leiomyosarcomas exhibit a significantly higher frequency of allelic loss (52%) compared with benign leiomyomas (18%) and smooth muscle tumors of uncertain malignant potential (21%) [41].

## 8. Therapeutic Considerations: Molecular Targeted Therapy

Development of targeted anticancer drugs is the direct result of knowledge of the molecular profile of endometrial neoplasms. Drug targets may focus on genes that affect apoptosis, signal transduction, epigenetic modification, drug resistance, protein folding and degradation, cell cycle progression, hormone receptor activity, and angiogenesis [4]. The drugs that comprise targeted therapy include small molecular weight inhibitors, monoclonal antibodies, antisense and gene therapy [61]. At this time, essentially only endometrial carcinomas have been tested with targeted therapy. Carcinosarcomas and endometrial stromal sarcomas are relatively uncommon neoplasms, and there has little experience with specific therapies for these tumors, though there is definitely future potential.

### 8.1. mTOR Inhibitors

The phosphatidylinositol-3-kinase (PI3K)-serine/threonine kinase (AKT)-mammalian target of the rapamycin (mTOR) signaling pathway plays a central role in the regulation of cell growth, proliferation, and apoptosis. In in vitro studies, cells with *PTEN* inactivation in endometrioid carcinoma are sensitive to mTOR inhibitors, since the loss of *PTEN* leads to constitutive activation of downstream components, which in turn up-regulates mTOR activity [62]. Potential therapies targeting the mTOR pathway include the mTOR inhibitors temsirolimus (CCI-779), everolimus (RAD001), and deforolimus (AP23573) [4]. In a phase II study of temsirolimus activity in patients with advanced or recurrent endometrial cancer, 5 of 19 (26%) evaluable patients had a partial response and 12 (63%) had stable disease [62, 63]. In addition to mTOR inhibitors, other agents targeting components of the mTOR-AKT-PI3K-PTEN pathway have also been developed, including enzastaurin (a PI3K inhibitor) and triciribine (an AKT inhibitor) [4].

### 8.2. EGFR Inhibitors/Anti-HER2/neu

Epidermal growth factor receptor (EGFR) family members (ERBB1 (EGFR or HER1), ERBB2 (HER2/neu), ERBB3 (HER3), ERBB4 (HER4)) are tyrosine kinase receptors that are activated by binding to epidermal growth factor (EGF)-like growth factor, leading to downstream phosphorylation or dephosphorylation of signaling molecules that involved in cell cycle and apoptosis [63]. Sixty to 80% of endometrial carcinomas overexpress EGFR [4]. In addition, EGFR expression has been described in approximately 70% of endometrial stromal sarcomas [41]. EGFR overexpression has been reported in high grade carcinomas with deep myometrial invasion, positive peritoneal washings and poor survival [61, 63]. The anti-EGFR agents result in down regulation of the MAPK and PI3K/AKT signaling pathways. However, the anti-tumor activity has been described in a minority of the patients treated. Antagonists to EGFR include small molecule tyrosine kinase inhibitors (gefitinib, erlotinib, and lapatinib) and the anti-EGFR monoclonal antibody cetuximab [62]. Experimental observation data have been shown that EGFR inhibitors could be more effective in endometrioid endometrial carcinoma than in uterine papillary serous carcinoma [63].

As described above, *HER2/neu* gene overexpression and amplification have been found in up to 80% of nonendometrioid endometrial carcinoma, and in 10–30% of endometrioid endometrial carcinoma. The usage of trastuzumab, a monoclonal antibody directed against HER2/neu, has been tested in endometrial carcinomas. Villella's group found 5 out of 19 (26%) patients with papillary serous carcinoma showed HER2/neu overexpression. One of 5 positive HER2/neu patients with advanced disease treated with trastuzumab achieved a complete response and a second patient's disease stabilized [63]. However in another study, Gynecologic Oncology Group (GOG)-0181-B, investigated trastuzumab in advanced, recurrent, or persistent endometrial cancer, and its preliminary results showed minimal activity, even in cancers with high overexpression of HER2/neu [4]. Several other monoclonal antibodies targeting members of the ERBB/HER family, including pertuzumab, cetuximab, and panitumumab, are currently being investigated [4].

### 8.3. Antiangiogenics

Vascular endothelial growth factors (VEGF) expression has been found in 56–100% of endometrial carcinomas [63] and has been correlated with high histologic grade, deep myometrial invasion, angiolymphatic invasion, nodal metastasis, and short disease-free survival [63, 64]. VEGF, particularly VEGF-A, plays a key role in angiogenesis and increased permeability of tumor-associated blood vessels. Monoclonal antibodies targeting VEGF, bevacizumab and sorafenib, have been developed. Kamat and coworkers [64] injected Ishikawa cell line into uterine horn of nude mice in one group and Hec-1A cell lines in the other group and treated the mice with docetaxel and/or bevacizumab. The combination of both agents had a greater efficacy in tumor growth inhibition than a single agent. Currently, GOG-229-E is being studied in a phase II trial of single agent bevacizumab in patients with recurrent endometrial carcinoma [64].

## 9. Conclusion

Knowledge of the molecular profiles of endometrial neoplasms assists in the diagnosis, prognosis, and treatment of endometrial neoplasms. Endometrial carcinoma can be broadly divided into two categories based on clinical behavior and morphologic phenotype, with good correlation to the molecular findings. Type I endometrial carcinoma represents an estrogen-related tumor, which usually arises in the setting of endometrial hyperplasia and have good prognosis. They are associated with a number of well-described genetic alterations including mutations of *PTEN, KRAS*, *β*-catenin, *PIK3CA*, and inactivation of DNA mismatch repair. Targets for molecular therapy in endometrial carcinoma include agents that inhibit components of the AKT-PI3K-PTEN pathway. Type II endometrial cancers are not estrogen-related and have poor prognosis. Mutations of *p53* are present in approximately 90% of this tumor type. Carcinosarcoma is considered to be a high-grade carcinoma with sarcomatous differentiation and a high frequency of *C-MYC* mutations and LOH of *p53*. The majority of endometrial stromal nodules and stromal sarcomas seem to originate from the abnormal *JAZF1/JJAZ1* gene fusion. The molecular biology of undifferentiated endometrial sarcomas is still not clearly delineated. In the near future; additional molecular studies should further elucidate the unclear pathogenesis and provide new targets for diagnosis and treatment.

## Figures and Tables

**Table 1:** Clinical and pathological characteristics of type I and type II endometrial carcinoma [1, 2, 4–13].

	Type I	Type II
Proportion of endometrial carcinomas	4/5	1/5
Menstrual status	Pre- and perimenopausal	Postmenopausal
Endocrine-metabolic disturbance	Present	Absent
Estrogen-associated	Yes	No
Background endometrium	Hyperplasia	Atrophy
Histological type	Endometrioid	Serous, clear cell
Tumor grade	Low	High
Depth of myometrial invasion	Superficial	Deep
Behavior	Stable/indolent	Progressive/aggressive

**Table 2:** Genetic alterations of type I and type II endometrial carcinomas, reported in percentages (references).

	Type I	Type II
*PTEN *inactivation	Up to 83% [1, 4, 11, 12]	11% [1, 2, 12]
*PIK3CA* mutation	26–36% [7, 9]	5% [7]
*KRAS* mutation	10–30% [1, 2, 4, 7–12, 17]	0–10% [2, 12]
*β*-catenin /*CTNNB1* mutation	14–44% [7, 8]	0–5% [1, 7, 10, 11]
Microsatellite instability	20–45% [1, 7–10]	0–11% [8, 9]
*p53* mutation	10–20% [1, 4, 6, 7, 10, 11, 13, 17, 18]	90% [1, 2, 4, 6, 7, 10–13, 17]
*HER2*/*neu* amplification	10–30% [1, 4, 10, 17]	18–80% [13]
*p16* inactivation	10% [1, 4, 7, 10, 11]	40–45% [4, 7, 10]
E-cadherin loss	10–20% [1, 4, 7, 10, 11]	60–90% [4]

**Table 3:** Typical immunoprofile of type I endometrioid carcinoma and type II serous carcinoma.

	Endometrioid carcinoma	Serous carcinoma
Estrogen and progesterone receptors	+	−
PTEN	−	+
*β*-catenin	+	−
p53	−	+
*HER2/neu*	−	+

+: positive result, −: negative result
